# Knockout mice are an important tool for human monogenic heart disease studies

**DOI:** 10.1242/dmm.049770

**Published:** 2023-03-21

**Authors:** Pilar Cacheiro, Nadine Spielmann, Hamed Haseli Mashhadi, Helmut Fuchs, Valerie Gailus-Durner, Damian Smedley, Martin Hrabĕ de Angelis

**Affiliations:** ^1^ William Harvey Research Institute, Queen Mary University of London, London EC1M 6BQ, UK; ^2^ Institute of Experimental Genetics, German Mouse Clinic, Helmholtz Center Munich, Munich 85764, Germany; ^3^ European Molecular Biology Laboratory-European Bioinformatics Institute, Hinxton CB10 1SD, UK; ^4^ Chair of Experimental Genetics, TUM School of Life Sciences, Technische Universität München, Freising 85354, Germany; ^5^ German Center for Diabetes Research (DZD), Neuherberg 85764, Germany

**Keywords:** Knockout mice, Monogenic heart disease, Multisystemic phenotypes

## Abstract

Mouse models are relevant to studying the functionality of genes involved in human diseases; however, translation of phenotypes can be challenging. Here, we investigated genes related to monogenic forms of cardiovascular disease based on the Genomics England PanelApp and aligned them to International Mouse Phenotyping Consortium (IMPC) data. We found 153 genes associated with cardiomyopathy, cardiac arrhythmias or congenital heart disease in humans, of which 151 have one-to-one mouse orthologues. For 37.7% (57/151), viability and heart data captured by electrocardiography, transthoracic echocardiography, morphology and pathology from embryos and young adult mice are available. In knockout mice, 75.4% (43/57) of these genes showed non-viable phenotypes, whereas records of prenatal, neonatal or infant death in humans were found for 35.1% (20/57). Multisystem phenotypes are common, with 58.8% (20/34) of heterozygous (homozygous lethal) and 78.6% (11/14) of homozygous (viable) mice showing cardiovascular, metabolic/homeostasis, musculoskeletal, hematopoietic, nervous system and/or growth abnormalities mimicking the clinical manifestations observed in patients. These IMPC data are critical beyond cardiac diagnostics given their multisystemic nature, allowing detection of abnormalities across physiological systems and providing a valuable resource to understand pleiotropic effects.

## Teaser

**Summary:** Systematic investigation of Genomics England PanelApp genes associated with monogenic forms of cardiovascular disease reveals that the International Mouse Phenotyping Consortium is a valuable resource for understanding pleiotropic gene effects.

## INTRODUCTION

Mouse models provide an invaluable resource to investigate the functionality of genes as well as contribute to the discovery of new genes involved in human disease (Baldridge et al., 2021). Genetically modified mice have markedly facilitated the understanding and characterisation of Mendelian disorders, providing insights into the disease mechanisms and the development of therapeutic options (Zarybnicky et al., 2021; Murillo-Cuesta et al., 2020). Reproducibility is vital in mouse disease modelling. Inappropriate breeding, animal husbandry and quality control can lead to irreproducible results. Having validated models, appropriate use of controls, rigorous experimentation and statistical standards for carrying out and reporting mouse phenotypic procedures are essential to strengthen the translational impact of model organisms (Justice and Dhillon, 2016; Lloyd et al., 2015). However, translation of phenotypes across different species presents some challenges. It is not always straightforward to assess whether a statistically significant abnormal phenotype, i.e. some deviation from normal morphology, physiology or behaviour (Robinson and Webber, 2014), detected in knockout mice will be observed when loss-of-function (LoF) mutations for the orthologue gene occur in humans (Spielmann et al., 2022). Conversely, knockout mice do not necessarily replicate the phenotypes observed in humans due to potential differences in biology, genetic background or alternative pathways; some of these limitations in mouse models have previously been discussed (Pound and Ritskes-Hoitinga, 2018; Elsea and Lucas, 2002). Phenotypes of adult animals are defined during development, so understanding the molecular evolution of phenotypic traits in mammals, including mice, is important to understand trait discrepancies (e.g. non-detectability) between species (Cardoso-Moreira et al., 2019).

The International Mouse Phenotyping Consortium (IMPC) is a collaborative, international programme that aims to generate and phenotype null mutants for every protein-coding mouse gene (Brown and Moore, 2012). It involves the creation of a repository and the implementation of standardised phenotypic assays to systemically characterise mouse strains across all organ systems. The standardised screens, which involve an ever-increasing number of wild-type mice, controlled for sex and other factors, are key for reproducibility. How well mouse models perform at mimicking disease phenotypes can be explored as a whole (Cacheiro et al., 2019) or focusing on specific types of disorders (Brommage and Ohlsson, 2019; McGraw et al., 2017). The IMPC is an evolving and frequently updated resource, with an increasing number of mouse knockouts and corresponding phenotype data points captured by data releases that provide stable and versioned reference sets of analysed data. At the time of the present analysis (IMPC Data Release 16.0), it contained 8093 phenotyped mouse genes and 93,235 phenotype calls (Groza et al., 2022).

Here, we systematically investigated the phenotypic abnormalities observed in the knockout mouse orthologues to human genes with evidence of cardiovascular function in humans. To this end, we used a set of Mendelian disease-associated genes from expert-curated gene panels used in clinical diagnostics from Genomics England PanelApp (Martin et al., 2019). We focused our analyses on a set of genes involved in monogenic forms of cardiomyopathy, cardiac arrhythmia and congenital heart disease showing clinical manifestations in humans that could be potentially captured by targeted IMPC assays, including electrocardiogram (ECG), transthoracic echocardiogram (TTE), organ morphology, gross pathology, embryo gross morphology and primary viability assessment.

## RESULTS

According to gene panels related to human Mendelian disorders, a total of 153 genes in the Genomics England PanelApp (Martin et al., 2019) have a high level of evidence for association with human cardiomyopathy, cardiac arrhythmia or congenital heart disease. For 151, we found the corresponding one-to-one mouse orthologue, and, out of these, 58 entered the IMPC phenotyping pipeline, with 57 genes showing at least one abnormal phenotype when knocked out in mice. These included preweaning lethality and/or effects on various physiological systems in the young adult (9-16 weeks) mice. For many genes, both were observed. We found 59.6% (34/57) of the homozygous knockouts to be lethal (absence of live null homozygous pups passing the weaning stage) and 15.8% (9/57) to be subviable (<12.5% live homozygous pups) in a primary viability assessment. For the non-viable (lethal and subviable) lines (43/57, 75.4%), the heterozygous knockouts entered the early adult phenotypic pipeline in which 69.8% (30/43) were found to be phenodeviants. All the homozygous viable lines (14/14) showed abnormalities in a broad range of physiological systems.

### Lethality in cardiac genes

**Fig. 1.**
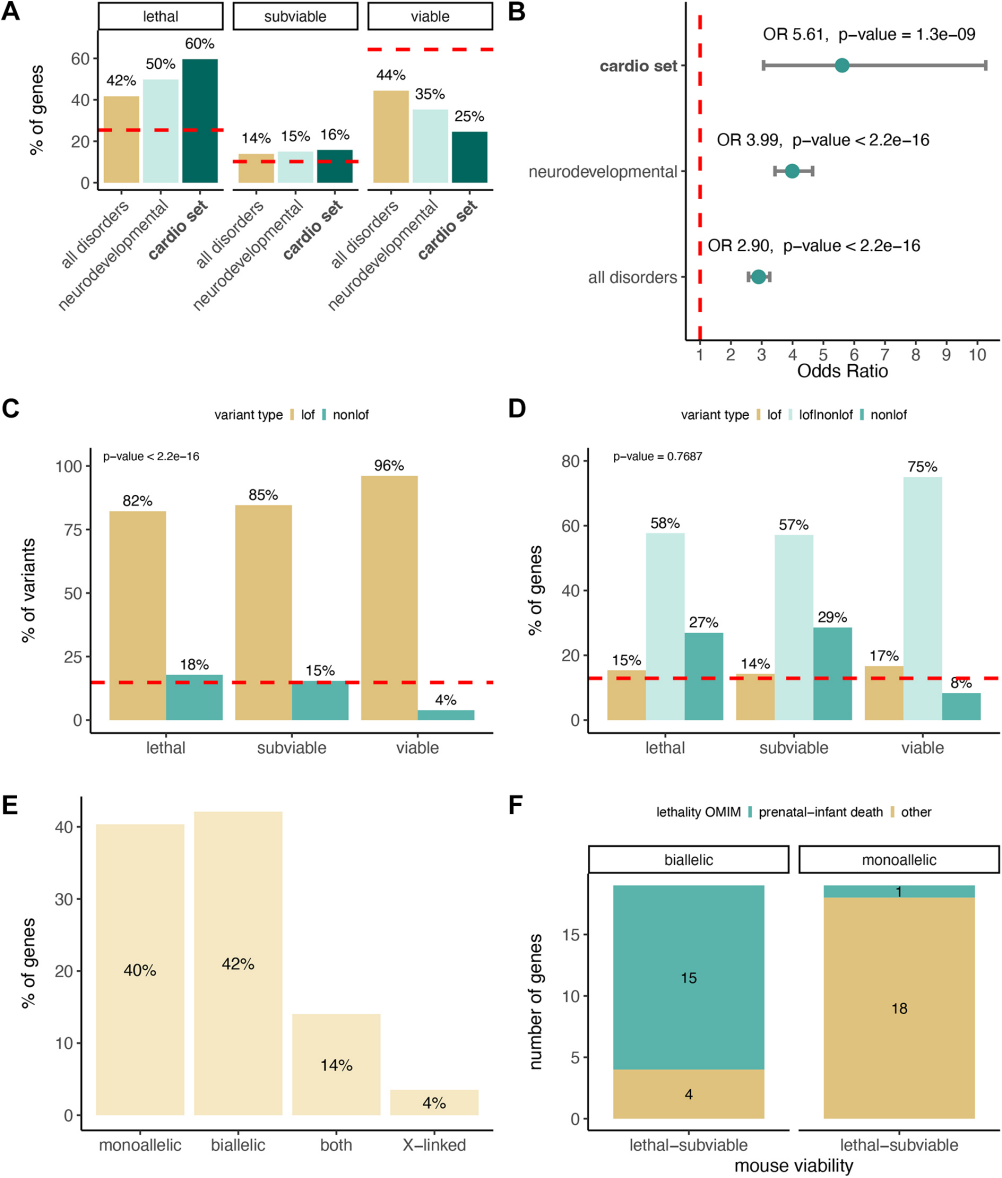
**Viability of the mouse orthologues of Mendelian cardiovascular disease genes.** (A) Percentage of lethal, subviable and viable lines for different sets of disease-associated genes. In the investigated cardiovascular set of genes, 60% of the lines were homozygous lethal with respect to 25% for the entire set of genes that have undergone viability assessment (dashed red line). Number of lines: lethal, *n*=34; subviable, *n*=9; viable, *n*=14. (B) The association between Mendelian disease genes and lethality in mice is statistically significant. Cardiovascular disease-associated genes have 5.6-fold higher odds of being lethal in the mouse primary viability assessment. ORs computed by unconditional maximum likelihood estimation and 95% CIs using the normal approximation, with the corresponding BH-adjusted *P*-values for the Fisher's exact test. (C) LoF variants are predominant in ClinVar, with the total number of LoF pathogenic variants reported for the cardiovascular genes investigated being significantly higher among genes viable in the International Mouse Phenotyping Consortium (IMPC) primary viability screen. The dashed red line indicates the percentage of non-LoF variants for the entire set of ClinVar variants investigated. (D) 27% of the cardiovascular genes showing lethality in mice have only non-LoF variants (mainly missense) reported as pathogenic in ClinVar. This percentage decreases to 8% for those genes viable in mice. The dashed red line indicates the percentage of genes with only non-LoF variants for the entire set of ClinVar variants investigated. Comparisons across viability categories were performed using two-sided Fisher's exact test. (E) Distribution of the set of genes investigated based on associated allelic requirement/mode of inheritance, showing a similar proportion of monoallelic and biallelic genes. (F) Most of the cardiovascular biallelic genes that are lethal/subviable in the knockout mouse show records of prenatal to infant lethality in human. This is not observed in the set of monoallelic genes. BH, Benjamini–Hochberg; CI, confidence interval; LoF, loss-of-function; OMIM, Online Mendelian Inheritance in Man; OR, odds ratio.

The percentage of lethal (and subviable) lines among the set of cardiovascular genes (43/57, 75.4%) compared to 35.03% for the total number of lines with viability data reflects how essential these genes are for organism development (Fig. 1A). Cardiovascular disease-associated genes have a 5.6-fold increase in the odds of being lethal/subviable in the mouse compared to 4-fold for neurodevelopmental disorder genes (Fig. 1B). When investigating lethality in human, 46.5% (20/43) of the lethal and subviable cardio genes in the mouse have also been associated with prenatal, neonatal or infant death up to 1 year of age according to Online Mendelian Inheritance in Man (OMIM) clinical records (Amberger et al., 2019). If we include childhood mortality (1-10 years of age), this percentage increases to 55.8% (24/43). Conversely, 28.5% (4/14) of the viable genes in mouse show records of neonatal/infant death in human. When we investigated potential sources of discrepancy in lethality between mouse and human, we found a higher percentage of (total) non-LoF pathogenic variants reported in ClinVar (Landrum et al., 2020) among the set of lethal/subviable genes in the mouse compared to the viable categories (*P*<2.2×10^−16^) (Fig. 1C). When investigating the presence of pathogenic LoF and non-LoF variants at the gene level, we also observed a lower percentage of genes with only non-LoF (mainly missense) variants reported, although the differences between viability categories are not statistically significant (Fig. 1D). In the studied set of knockout mice, the phenotype is invariably the result of a null – LoF – mutation.

Out of the 153 diagnostic grade genes associated with cardiomyopathy, cardiac arrhythmias and congenital heart disease in humans, 54 (35%) are biallelic (autosomal recessive; AR), 68 (44%) are monoallelic (autosomal dominant; AD), 21 (14%) are both biallelic and monoallelic, and 10 (7%) are X-linked. For the subset of these genes with IMPC mouse data available, we found a slight shift in the percentages of biallelic forms: 24 (42%) compared to 23 (40%) are monoallelic, eight (14%) are both biallelic and monoallelic, and two (4%) are X-linked (Fig. 1E). Interestingly, we did not observe differences in viability when we compared AR and AD disease-associated genes: for 19/24 (79%) AR disease genes and 19/23 (83%) AD genes, the mouse was homozygous lethal or subviable, respectively (*P*=1). A curation of OMIM clinical records indicates that 15/19 (79%) AR and homozygous mouse lethal genes have records of prenatal to infant lethality in humans, whereas this is true for only 1/19 (5%) AD and homozygous knockout lethal genes (Fig. 1F).

### Phenotype abnormalities

**Fig. 2.**
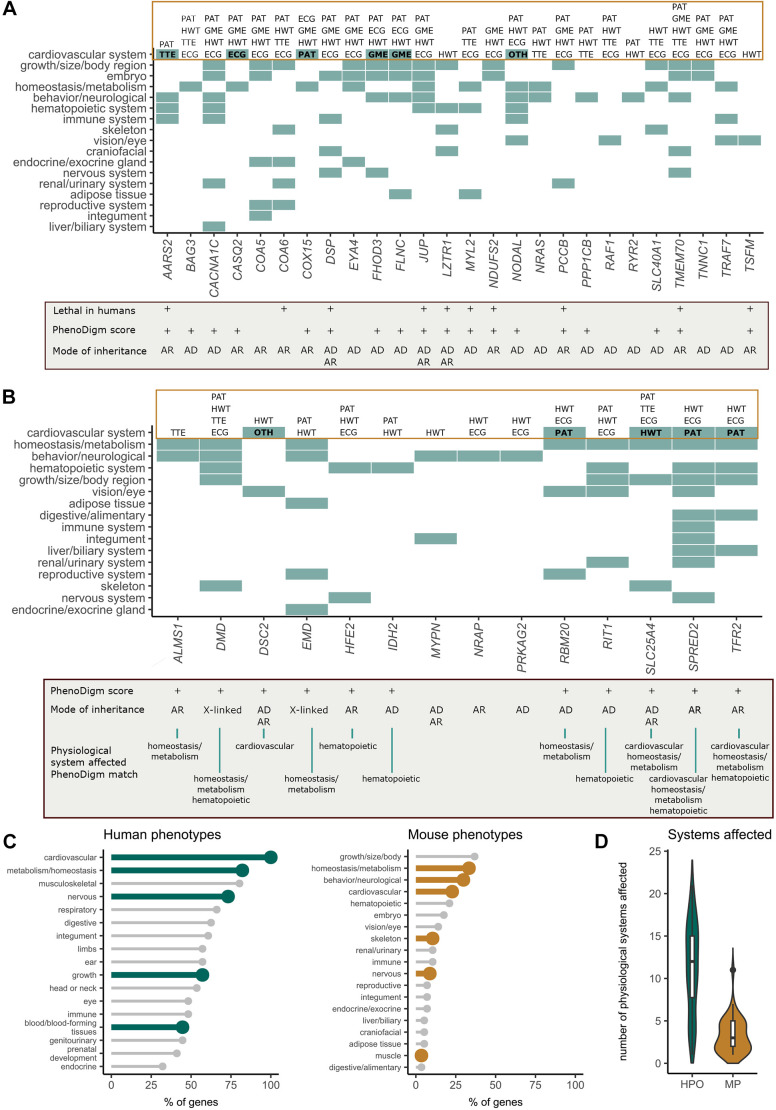
**Phenotypes observed in the mouse orthologues of Mendelian cardiovascular disease genes.** (A) IMPC embryo and heterozygous knockout mouse phenotypes for lethal lines. (B) IMPC homozygous knockout mouse phenotypes for viable lines. For genes in A and B, the physiological systems showing abnormal phenotypes are shown. For the cardiovascular system phenotypes, the phenotyping procedures that have been successfully performed are indicated at the top of the figure; those where a significant phenotype association was found are highlighted in bold. For the lethal lines, those genes for which early lethal phenotypes in humans have been reported are indicated, as well as those genes with positive PhenoDigm scores. For the viable lines, the physiological systems affected that are shared among patients and the knockout mice as captured by the PhenoDigm algorithm are highlighted. Additionally, two subviable genes, *ALPK3* and *DSG2*, showed cardiac abnormalities in the knockout mouse. (C) Human and mouse physiological systems associated with the set of cardiovascular genes according to HPO and IMPC MP annotations. Cardiovascular phenotypes are reported for 100% of the genes in humans; only 23% show a cardiac abnormality in the mouse. (D) The distribution of the number of physiological systems affected per gene illustrates the multisystemic nature of the associated disorders. AD, autosomal dominant; AR, autosomal recessive; ECG, electrocardiography; GME, gross morphology (embryo); HPO, human phenotype ontology; HWT, heart weight; MP, mammalian phenotype ontology; OTH, other, corneal vascularization; PAT, gross pathology and tissue collection; TTE, transthoracic echocardiography. The complete set of genes and annotations is available at doi:10.5281/zenodo.7476632.

A full description of the significant phenotypes other than lethality, i.e. phenotypes for the heterozygous knockout in homozygous knockout lethal lines and the homozygous knockouts for the viable lines, is shown in Fig. 2A and B, respectively. The majority of lethal lines (26/34, 76.4%) show an abnormal phenotype in heterozygous viable adult mice. The phenotypes observed in the embryo and early adult mice cover the whole range of physiological systems, and multisystem phenotypes are common (21/26, 80.8%) (Fig. 2A). Despite only six mouse models displaying cardiac abnormalities in the homozygous embryo or heterozygous early adult IMPC mice, abnormalities in other physiological systems mimicking some of the clinical phenotypes observed in patients were captured. This is reflected by the PhenoDigm scores (Smedley et al., 2013) (see Materials and Methods) used to compute the phenotypic similarity between the mouse model [mammalian phenotype ontology (MP) terms] and the human disease phenotypes [human phenotype ontology (HPO) terms]. A PhenoDigm percentage score greater than 0 implies at least one HPO–MP match. Based on these scores, 69.2% (18/26) of the homozygous lethal lines showing phenotypic abnormalities in the heterozygous model are able to partially recapitulate the phenotypic manifestations observed in patients.

All of the viable lines showed at least one physiological system affected, with all but three genes (11/14, 78.6%) showing abnormal phenotypes in different organ systems. Only five of 14 genes showed some variation of cardiovascular phenotypic abnormality. Abnormalities in other physiological systems, e.g. homeostasis and/or hematopoietic system reported for the associated disorders, were also recorded in the mutant mice. For three genes (*MYPN*, *NRAP* and *PRKAG2*), no overlapping abnormal phenotypes have been found between mouse and human. For some genes, the phenotyping procedures aimed at identifying abnormal cardiovascular phenotypes in the mouse have not yet been performed, e.g. *IDH2* and *MYPN* with no ECG or TTE data (Fig. 2B).

If we compare the phenotypes observed in patients according to HPO annotations with the abnormal phenotypes identified in the knockout mice, some of the organ systems more frequently affected are shared between the two organisms: cardiovascular, metabolism/homeostasis, musculoskeletal and nervous system abnormalities (Fig. 2C)*.* Multisystemic phenotypes are predominant both in mouse and humans (Fig. 2D). Based on HPO annotations for these genes, only four genes have associated abnormal phenotypes restricted to the cardiovascular system (*JPH2*, *TNNI3K*, *COA5* and *PKP2*), being the median number for this set of genes of 12 physiological systems, which illustrates the multisystemic nature of the associated disorders. The complete set of genes and annotations is available at doi:10.5281/zenodo.7476632.

### Phenotyping coverage

**Fig. 3.**
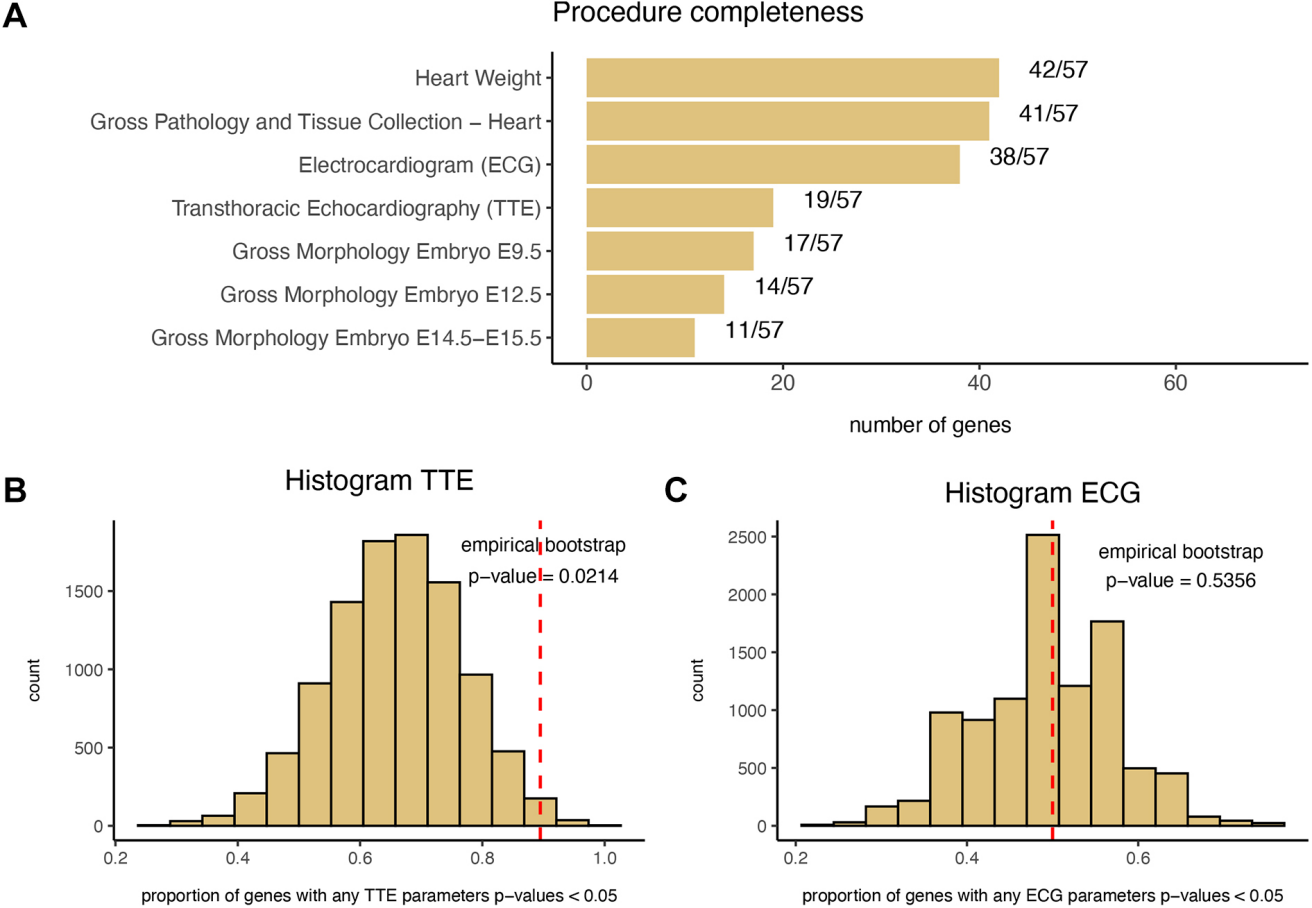
**Phenotyping procedures aimed at capturing cardiac abnormalities.** (A) Percentage of the IMPC phenotyping lines by procedure (or trait). (B,C) Empirical bootstrap distribution of the proportion of genes with significant nominal *P*-values for any of the parameters evaluated through the TTE (B) and ECG (C) procedures. The empirical *P*-values are obtained by computing the number of iterations (out of 10,000) with a proportion of genes greater or equal to the observed proportion in the gene group of interest as described in the Materials and Methods (‘Bootstrap approach’) section.

The IMPC *in vivo* ECG and *in vivo* TTE constitute the main phenotyping procedures aimed at capturing structural and functional heart anomalies of the heart in mice. ECG peak and interval detection are used to capture electrical conduction problems, whereas TTE recordings are performed to capture morphological and functional phenotypes of the left ventricle at end-systole and end-diastole. *Ex situ* iodine-contrast high-spatial-resolution microcomputed tomography imaging of embryo hearts is applied at different developmental stages to identify morphological cardiac abnormalities or developmental delays. Gross pathology and tissue collection allow the detection and recording of abnormal external findings and macroscopic alterations in the heart, including heart weight. The IMPC phenotyping pipeline is performed across different centres with slightly variable coverage. Nine IMPC centres measure ECG and six centres perform TTE recordings at 12 weeks of age in mice. *Ex situ* imaging of the embryo heart is solely applied to homozygous lethal or homozygous subviable single-gene knockouts to identify structural heart defects. No embryo imaging was performed on viable knockout lines so embryo data were available for only a subset of the total knockout genes used in this study. This results in a situation where the current phenotyping may have not yet covered the relevant physiological systems affected in patients. Consequently, cardiac phenotype coverage is gaping, or the specific test cannot be included anymore; herein, not all lines can be evaluated for the complete set of cardiovascular parameters. Fig. 3A shows the coverage of the main procedures aimed at capturing cardiovascular phenotypes in the set of selected genes.

### Bootstrap analysis

For each phenotyping procedure, the IMPC data are processed by different statistical methods to identify genotype–phenotype associations by comparing knockout mice to wild-type mice of the same background strain. Given the number of tests performed, a pre-set and conservative significance threshold (0.0001) for Type I error has been established. This cut-off is used to identify phenodeviants and results in an MP term being associated with a knockout line. Given the observed depletion of cardiovascular phenotypes, we further investigated the raw statistical results to check the *P*-values for the parameters evaluated through the two main TTE and ECG procedures. Bootstrap was applied to assess whether the proportion of nominally significant *P*-values (<0.05) among the selected genes was higher than expected (see Materials and Methods). The proportion of genes with at least one significant parameter (*P*<0.05) in the set of cardiovascular gene pairs with TTE procedures is 0.89 (17/19) compared to 0.66 for the entire set of genes with TTE procedures (empirical bootstrap *P*=0.0214). Applying the same procedure to genes with ECG parameters, the proportion of *P*-values <0.05 in the set of genes of interest is 0.53 (20/38) compared to 0.49 for the entire set of genes with ECG procedures (empirical bootstrap *P*=0.5356) (Fig. 3B,C). This suggests that there is an enrichment for nominally significant *P*-values for the TTE procedure.

### Extended analysis of genes with moderate evidence

**Fig. 4.**
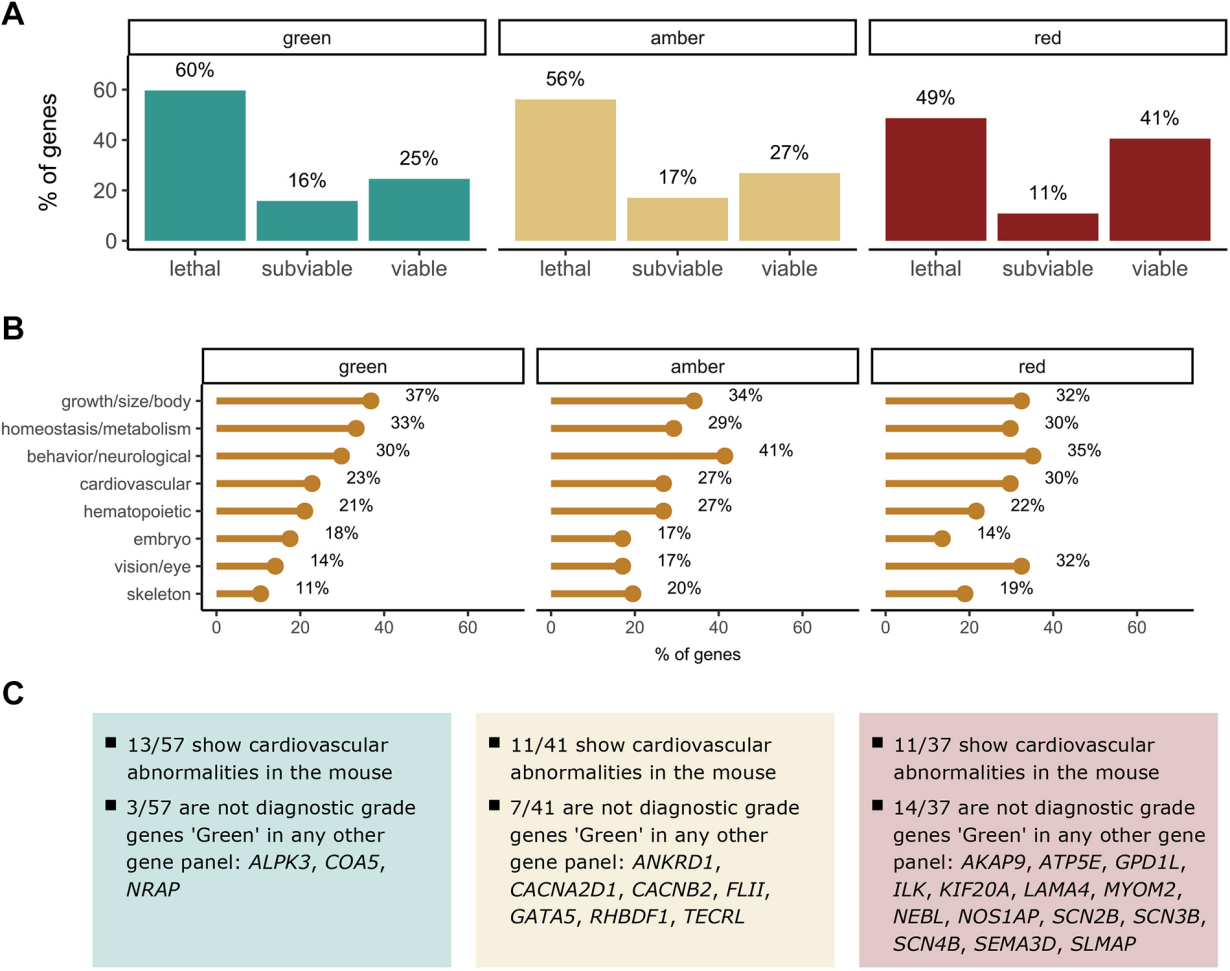
**Additional PanelApp genes based on the level of evidence for the gene-phenotype association.** (A) Percentage of lethal, subviable and viable lines for different sets of cardiovascular genes based on the PanelApp rating that is used for genome interpretation. (B) Percentage of genes in each category showing abnormal phenotypes for the main physiological systems affected. (C) Most of the genes labelled as ‘green’ are also considered diagnostic-grade genes in other gene panels (95%), compared to 62% of genes classified as ‘red’. Number of genes: green, *n*=57; amber, *n*=41; red, *n*=37.

The previous analysis was expanded to include genes for which the level of evidence for their association with the phenotype targeted in the gene panel from PanelApp is moderate or low (‘amber’ and ‘red’). This added 90 additional genes labelled as amber and 104 genes classified as red. It is worth mentioning that a single gene may have a different status in several panels, i.e. *SLC25A4* is rated as ‘green’ in the ‘Cardiomyopathies – including childhood onset’ panel and as red in the ‘Hypertrophic cardiomyopathy – teen and adult’ panel. Out of 194 extra genes, 78 have entered the IMPC phenotyping pipeline. A summary of viability information and phenotypic abnormalities observed in the knockout mouse for these genes is shown in Fig. 4. Interestingly, an even higher percentage of amber and red genes, compared to green genes, show cardiovascular abnormalities in the mouse, although the differences between genes based on the level of evidence for the disease association are not significant (viability, *P*=0.239; cardiovascular phenotype, *P*=0.726).

## DISCUSSION

### Lethal phenotypes

Genes resulting in a preweaning lethality phenotype in the mouse are known to be enriched in human disease; however, the percentage of lethal/subviable lines found among the set of cardiovascular genes investigated (75.4%) is exceptionally high compared to that of the entire set of Mendelian disease-associated genes (55.6%) or neurodevelopmental disease genes (64.7%) (Dickinson et al., 2016; Cacheiro et al., 2020). By identifying early lethal phenotypes through curation of OMIM records, 50.9% of the associated disorders also had clinical reports of prenatal, neonatal, infantile or childhood lethality. The discrepancy between the genes showing homozygous LoF lethality in the mouse but not in human disease may be explained by factors such as the following: (1) 97.5% of the IMPC mutant lines are derived from the C57BL/6 substrain genetic background, compared to a higher polygenic background found in humans that may affect the penetrance and/or severity of some cardiac phenotypes and impact the phenotypic associations observed in more genetically diverse patients (Fahed et al., 2020); (2) genes with monoallelic (heterozygous) variants account for 40.3% (23/57) of the genes included in this study, and biallelic (homozygous) variants could lead to embryonic lethality in humans; reports of prenatal lethality may be underestimated in current disease annotation databases with the homozygous variant not documented in humans; (3) even for biallelic forms of disease, a wide range of phenotypic outcomes in terms of severity could be expected, with the difficulty to establish a molecular diagnosis when prenatal lethality occurs; and (4) particularly among the genes showing mouse lethality, there is a non-negligible percentage of genes (27%) for which only non-LoF variants have been classified as pathogenic, which could be mildly deleterious and thus associated with less severe phenotypes (Engwerda et al., 2021). This higher percentage of genes with only missense variants observed in patients for genes that are lethal in the mouse compared to viable genes may again indicate that LoF variants are not seen in the population because they lead to prenatal death in humans. Conversely, for the set of viable genes, LoF variants may not lead to early death but to less severe phenotypes, whereas missense variants could have a mild or negligible impact on protein function.

### Detecting abnormal phenotypes through ECG and TTE

Despite considerable differences in heart size and conduction rate, the development of the four-chambered heart and cardiac anatomy is remarkably similar in mice and humans (Wessels and Sedmera, 2003). Hence, single-gene-knockout mouse lines with abnormal electrocardiography and/or echocardiography are well-established model systems for evaluating genotype–phenotype associations that may cause or contribute to human heart disease. *In vivo* ECG and TTE, two state-of-the-art diagnostic procedures (British Heart Foundation), have here enabled us to recapitulate phenotypes in mice known to be manifested in congenital monogenic forms of cardiac arrhythmias and cardiomyopathies in neonates and children. There is a variable coverage for certain phenotype assays, including ECG and TTE (a challenge of the high-throughput and multicentre approach), and therefore not all mouse lines undertake the same number of tests. This gap can partially justify the rather marginal abnormal cardiovascular phenotype detection rate of IMPC for the known cardiac genes described in PanelApp (Fig. 2A,B). This incompleteness is certainly a reason for lack of phenotypic recapitulation in the mouse. For example, for the 14 viable lines, nine did not show any cardiovascular phenotype, but for four lines neither ECG or TTE was performed and for 11 lines TTE was not performed, which is required to detect the types of morphological and functional heart phenotypes described in human patients (e.g. cardiomyopathy). Furthermore, in high-throughput phenotyping as pursued by the IMPC, the detectability of differences in the interval lengths of ECG parameters and thus initial indications of abnormal gene function in the innervation of the heart were recorded, but not arrhythmias.

Another issue raised during our analyses is whether the stringent thresholds to detect abnormal phenotypes through the ECG and TTE are obscuring some true effects. Multiple testing correction is required to avoid inflated false-positive rates in the detection of the phenotype associations, but the enrichment of unadjusted *P*-values for the TTE parameters among the set of cardiovascular genes suggests that this particular procedure could be reviewed to investigate alternative cut-offs (e.g. 8/19, 42% genes with TTE data would have a significant abnormal heart phenotype with an alpha significance level of 0.005). The focus of the IMPC is to unlock, through systematic genotyping in mice, a landscape of previously unknown or poorly described genes that play a role in congenital monogenic heart disease and have great potential for translation.

### Capturing multisystemic phenotypes

Cross-species phenotypic similarity algorithms allow for automated analysis of phenotype information and evaluation of mouse models for known disease–gene associations. PhenoDigm (Smedley et al., 2013) relies on the entire set of mouse and disease annotations, using all the pairwise mouse and human phenotype comparisons and thus identifying partial matches, i.e. a shared abnormal phenotype in one particular physiological system.

Neuromuscular disorders are commonly associated with cardiomyopathy, e.g. Emery–Dreifuss muscular dystrophy and Duchenne muscular dystrophy, with skeletal muscle phenotypes showing an earlier age of onset (McNally and Mestroni, 2017). Similarly, specific mitochondrial and storage diseases and inborn errors of the metabolism are also associated with cardiomyopathy (McNally and Mestroni, 2017; Lloyd et al., 2017). Congenital heart disease is also associated with a higher risk of neurodevelopmental delay (Homsy et al., 2015). Unsurprisingly, these are the physiological systems more commonly affected along with cardiovascular abnormalities both in patients and knockout mice, indicating that the unbiased nature of the IMPC pipeline is able to capture this wide range of co-occurrent phenotypic outcomes, even when specific cardiac abnormalities were not detected. It is important to note the variable spectrum of clinical features reported in patients that are summarised by one set of HPO-associated terms. Given this and other factors involved, e.g. LoF versus non-LoF variants or the complex nature of the genotype–phenotype relationship, it is not unexpected to find that only a subset of all the clinical manifestations observed in humans is mimicked in the mouse.

For the genes in the selected panels with IMPC data, only three are not included as green genes in any other panel (*ALPK3*, *COA5*, *NRAP*). *RYR2*, *TNNC1*, *CALM2*, *CASQ2*, *FHOD3*, *RBM20*, *TMEM43*, *DSG2* and *MYL2* are also included in the sudden cardiac death panel. The remaining 44 genes are considered as diagnostic genes in several other panels, predominantly neurology and neurodevelopmental disorders (23), metabolic disorders (22), dysmorphic and congenial abnormal syndromes (6) and endocrine disorders (5). Similarly, based on HPO annotations, only a small percentage of the studied genes (7%, 4/57) have associated abnormal phenotypes restricted to the cardiovascular system.

The IMPC resource and the data presented here are not without limitations. Owing to its high-throughput nature, the current pipeline focuses on phenotyping a minimal number of animals required to detect strong phenotypic effects (de Angelis et al., 2015). The phenotypic screens have been selected to facilitate modelling of human disease, but some key biological functions are not fully covered or studied in the heart (e.g. right ventricular cardiac diagnostics, blood pressure measurements), or in some cases only in centre-specific pipelines or pilot studies; thus, incomplete phenotype coverage across mouse lines for certain procedures is experienced. Further, only protein-coding LoF effects of single genes are characterised, and, importantly, the practical totality of IMPC lines is derived from one substrain genetic background, C57BL/6 and differences in the phenotypic outcomes of mutations from different genetic backgrounds could be observed given potential effects on penetrance and expressivity (Simon et al., 2013; Doran et al., 2016).

In summary, the analysis of the outcomes of a systematic phenotyping pipeline performed on knockout mice for a subset of genes involved in monogenic forms of cardiovascular disease uncovered an important finding of gene essentiality. A significantly higher number of lethal lines compared to other disease categories was identified, reflecting how this set of genes has an indispensable role in organism development. Specific cardiovascular phenotypes were, however, not identified in the majority of heterozygous knockout or the homozygous knockout viable lines, and different hypotheses were provided for this lack of recapitulation. The approach followed in the present study highlights the benefits of the multisystem nature of the phenotyping protocol, allowing the detection of abnormalities in several other physiological systems important to mimicking the clinical manifestations observed in patients, and is thus providing a useful transformative resource to clinicians. Here, we focused on a set of diagnostic-grade genes; however, for some of the genes, there is no evidence from unrelated families and/or enough functional evidence to validate the association with the observed phenotypes. The highly standardised high-throughput screening pipelines such as the IMPC contribute to generating a high volume of data and provide mouse models that can assist the prioritisation and validation of variants found in those genes. The identification of genes, mutations, and their mechanisms of action that cause and/or contribute to congenital heart disease in humans is far from complete, and alternative approaches for novel disease-gene discovery can benefit from this genotype–phenotype knowledge base (Spielmann et al., 2022).

## MATERIALS AND METHODS

### Monogenic cardiovascular disease gene selection

Genomics England PanelApp, a publicly available resource that contains expert curated gene panels for human Mendelian disorders, was used to identify genes of interest. Genes are categorised according to the level of evidence to support the gene–disease association. We retrieved green genes, i.e. genes with high confidence of being associated with disease, included in any of these three categories: ‘Cardiomyopathy’, ‘Congenital heart disease’ and ‘Cardiac arrhythmia’ (Genomics England PanelApp data accessed 23 November 2022) (Martin et al., 2019). A single gene can belong to multiple gene panels (Table S1). Subsequent analysis was limited to the subset of genes with mouse orthologues that have undergone the IMPC phenotypic pipeline (*n*=57).

An additional analysis was performed to include genes in the aforementioned panels labelled as amber (moderate evidence for the gene–disease association; it should not be used for genome interpretation) and red (not enough evidence for the gene–disease association; it should not be used for genome interpretation). This added 90 amber and 104 red genes, of which 41 and 37, respectively, have a one-to-one mouse orthologue that entered the IMPC phenotypic pipeline.

### IMPC mouse phenotyping procedures and data analysis

The IMPC has produced a standardised pipeline that all phenotyping centres follow. Detailed information on the standardised phenotyping pipelines and standard operational procedures (SOPs) is available through the Mouse Phenotyping Resource of Standardised Screens (IMPReSS). The IMPC relies on the Animal Research: Reporting of *In Vivo* Experiments (ARRIVE) guidelines, with all phenotyping pipelines designed to ensure that there are no welfare issues for a normal mouse and that the welfare of the animals is being routinely assessed (Karp et al., 2015). Data were obtained from knockout mice phenotyped under the viability, young adult (12 weeks of age) and embryonic phenotype pipelines. All mice were on the C57BL/6 background. Both sexes were analysed with a sample size of roughly 14 knockout mice (seven female, seven male), and wild-type mice were continually tested with the same protocols to produce baseline (normal) values. Details of mouse production including breeding, housing and husbandry, animal care and monitoring as well as study design, experimental procedures, sample size, exclusion and inclusion criteria can be found on the IMPC site.

All measurements from knockout mice are accompanied by corresponding data from matched wild-type controls. All these data are further annotated with metadata. For each phenotyping screen, the IMPC data are processed by applying Soft windowing (Haselimashhadi et al., 2020) implemented in the SmoothWin R package accompanied by OpenStats, an R package that provides a set of statistical methods to identify genotype–phenotype (MP term) associations by comparing knockout to wild-type mice (Haselimashhadi et al., 2021). The genotype effect is assessed using linear mixed models and under the setting of 0.0001 for the Type I error. The Soft windowing procedure assigns more weight to the set of control mice that were assessed proximally to the knockout mice. Batch effects are also considered in the modelling approach.

The IMPC strategy for the upcoming years is to prioritise mouse orthologues of human genes that are not well understood, with very limited knowledge about their function, i.e. the dark genome (Brown and Lad, 2019). IMPC welcomes gene nomination and prioritisation requests from the research community.

### Human phenotypes

Human phenotypes for the selected genes were retrieved using the HPO annotation files (Köhler et al., 2021), and through OMIM curations of the associated clinical records for evidence of early lethality (Amberger et al., 2019).

### PhenoDigm scores

The PhenoDigm algorithm (Smedley et al., 2013) computes individual scores for each HPO–MP phenotypic match, based on the proximity of the two terms in the overall cross-species ontology (Jaccard index; simJ) and the observed frequency of the phenotype in common from the entire set of disease and mouse annotations (information content; IC). The geometric mean of the IC and simJ was used to generate the HPO–MP pairwise score. The overall score, which is a percentage-based score, is the result of comparing the best and mean scores for all the pairwise HPO–MP comparisons relative to the maximum possible scores for a mouse model perfectly mimicking the disease phenotypes. A PhenoDigm percentage score greater than 0 implies at least one HPO–MP match.

### Variant functional annotations

Molecular consequences for the pathogenic variants reported for each gene were obtained from ClinVar. Only pathogenic variants according to clinical significance were considered for the analysis. Molecular consequence categories were classified as LoF (frameshift, nonsense, splice site) and non LoF (missense, non-coding RNA, near gene, untranslated region, synonymous, in-frame). Each gene was labelled as LoF if at least one LoF pathogenic variant was reported or as non-LoF if only non-LoF pathogenic variants were reported (Landrum et al., 2020). This terminology was adopted for simplification, e.g. missense variants may act through LoF mechanisms.

### Bootstrap approach

For the bootstrap approach, we retrieved the raw *P*-values for each gene–parameter pair from the ECG and TTE procedures. The empirical bootstrap aims to estimate parameters by iteratively sampling from the data (Efron and Tibshirani, 1993). We employed bootstrap to check whether the proportion of nominally significant *P*-values (<0.05) among the selected genes for the procedures of interest was higher than expected. To this end, the empirical bootstrap distribution of the proportion of pairs with nominally significant *P*-values was computed. A total of 10,000 iterations (equally sized without replacement and with a sample size equal to the number of genes with data for each procedure) are drawn from the pool of genes with raw *P*-values for the TTE and ECG procedures, and the proportion of genes in each iteration with at least one significant raw *P*-values is estimated. Based on the bootstrapped proportions, the empirical *P*-values are obtained by computing the number of iterations with a proportion of genes greater or equal to the observed proportion in the gene group of interest.

### Comparison across viability categories

For each disease category, odds ratios (ORs) were computed from a contingency table with the number of disease- and non-disease-associated genes for each of two viability categories (lethal/subviable and viable) for all those genes with IMPC data on viability according to IMPC Data Release 16. ORs were computed by unconditional maximum likelihood estimation and normal approximation (Wald) confidence intervals as implemented in the epitools R package. Two-sided *P*-values for the test of independence were computed using Fisher's exact test. The *P*-values that are represented in the text and figures were adjusted using Benjamini–Hochberg correction method (Fig. 1B). Other comparisons across viability categories were performed using Fisher's exact test (Fig. 1C,D). All the data analysis was performed using R.

### Ethical approval

All the IMPC international member institutes that breed mice and collect phenotyping data are guided by their own and individual ethical review panels, licensing and accrediting bodies, reflecting the national and/or geopolitical legislation under which they operate. Cardiovascular mouse phenotyping was carried out under the auspice of the following animal protocols: the German Mouse Clinic Helmholtz Zentrum München – German Animal Welfare Act; Medical Research Council Harwell – Animal Welfare and Ethical Review Body (AWERB); RIKEN Tsukuba Institute – The Animal Experiments Committee; The Centre for Phenogenomics – Animal Care Committee (ACC); Baylor College of Medicine – Institutional Animal Care and Usage Committee (IACUC); The Jackson Laboratory – IACUC; the University of California Davis – IACUC.

### Mouse phenotypic data

All IMPC phenotyping data are publicly available through the IMPC portal and FTP repository. Phenotyping procedures to capture the phenotypic manifestations potentially present in these disorders include primary viability, TTE (IMPC_ECH_001) and ECG (IMPC_ECG_001, IMPC_ECG_002) to assess the functional and morphological abnormalities of the heart, gross pathology, tissue collection and embryo gross morphology at different developmental stages [embryonic day (E)9.5, E12.5, E14.5-E15.5]. The complete list of viability outcomes and significant phenotypes corresponding to the IMPC Data Release 16 are publicly available to download and use (including the terms of use) (Dickinson et al., 2016).

## Supplementary Material

10.1242/dmm.049770_sup1 Supplementary information
