# Supplementary material for: Knockout mice are an important tool for human monogenic heart disease studies
Source: Dis Model Mech. 2023 Mar 21;16(5):dmm049770. doi: 10.1242/dmm.049770 (PMC10073007; doi:10.1242/dmm.049770)
Supplement: Supplementary information [file dmm-16-049770-s1.pdf]

Table S1. Gene panels from PanelApp included in the present study

Gene panel	Number of genes
Arrhythmogenic cardiomyopathy	10
Brugada syndrome	1
Cardiac arrhythmias	14
Cardiac arrhythmias - additional genes	1
Cardiomyopathies - including childhood onset	107
Catecholaminergic polymorphic VT	6
Dilated cardiomyopathy - adult and teen	32
Dilated Cardiomyopathy and conduction defects	35
Familial non syndromic congenital heart disease	25
Hypertrophic cardiomyopathy - teen and adult	22
Idiopathic ventricular fibrillation	1
Left Ventricular Noncompaction Cardiomyopathy	6
Long QT syndrome	9
Sudden death in young people	4
Total number of unique genes	153

Disease Models & Mechanisms • Supplementary information
